# Supplementary material for: Intraspecific Variability in Proteomic Profiles and Biological Activities of the Honey Bee Hemolymph
Source: Insects. 2023 Apr 6;14(4):365. doi: 10.3390/insects14040365 (PMC10142140; doi:10.3390/insects14040365)
Supplement: Supplementary file 1 [file insects-14-00365-s001.zip › insects-2255045-supplementary.pdf]

Supplementary materials

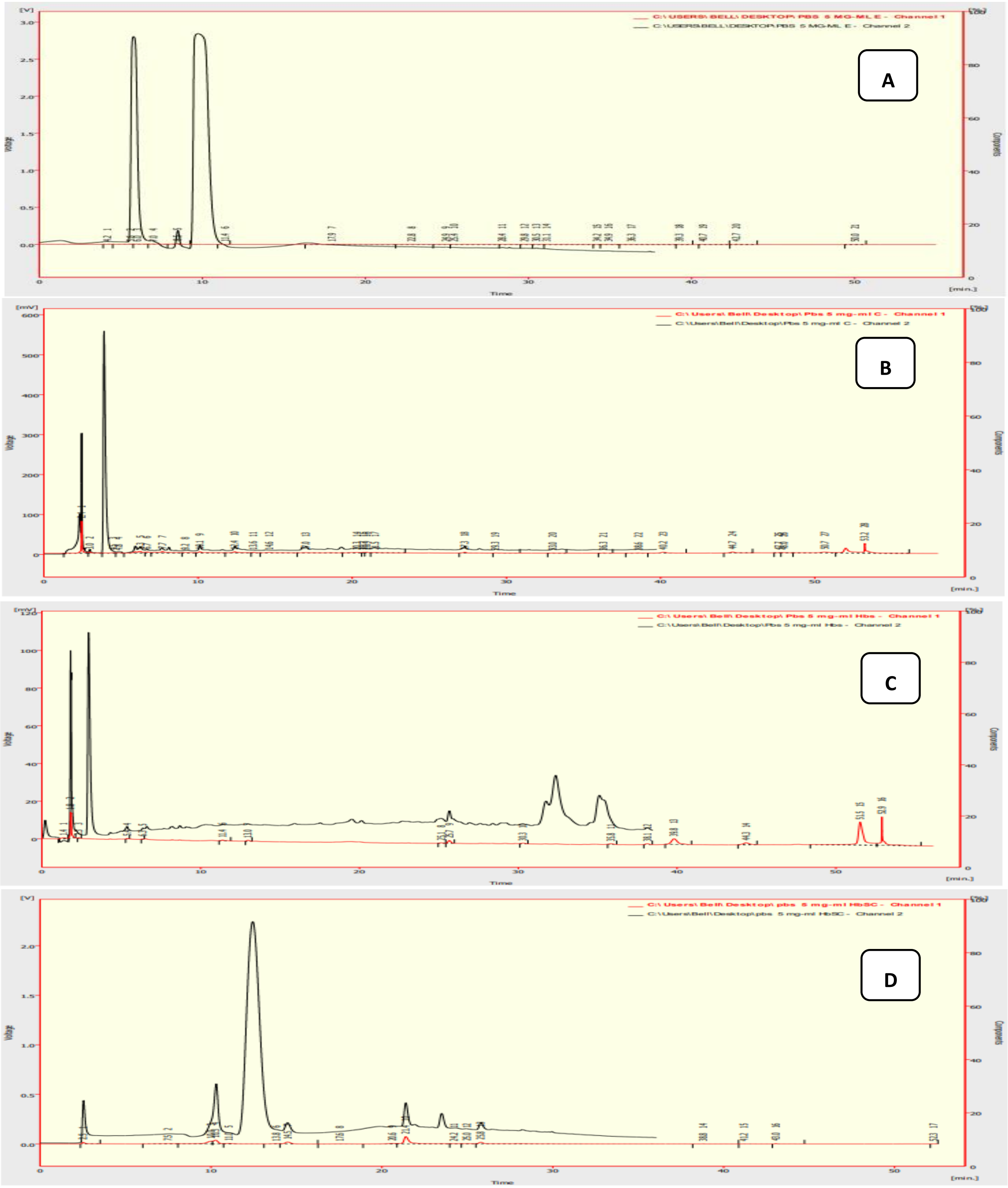

Figure S1. RP-HPLC chromatograms of the PBS dissolved honey bee hemolymph extracts where, (**A**): Bees from Port Said, (**B**): bees from Ismailia governorate, (**C**): bees from Suez governorate, (**D**): bees from Saint Catherine

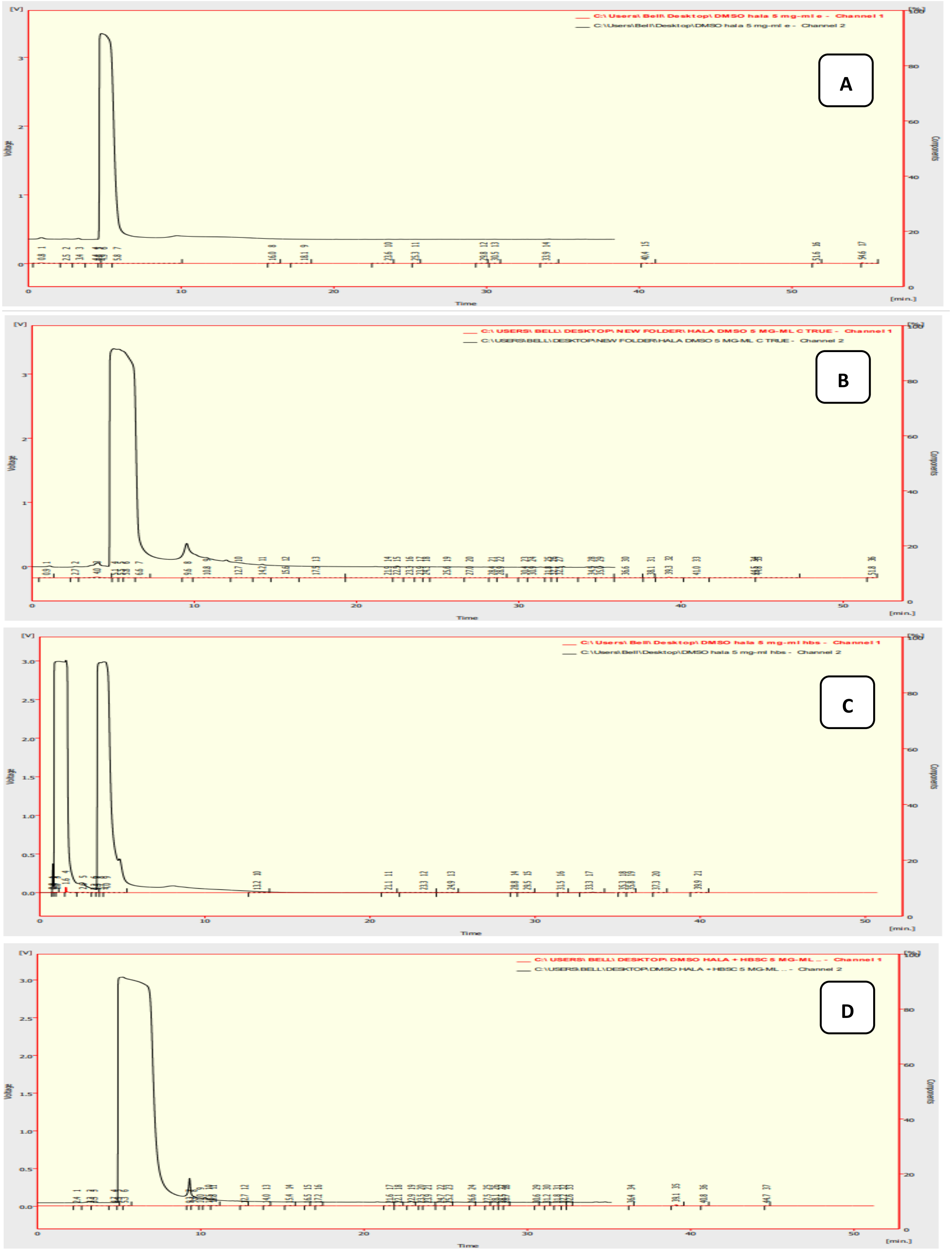

Figure S2. RP-HPLC chromatograms of the DMSO dissolved honey bee hemolymph extracts where, (**A**): Bees from Port Said, (**B**): bees from Ismailia governorate, (**C**): bees from Suez governorate, (**D**): bees from Saint Catherine
